# Impacts of land use, restoration, and climate change on tropical peat carbon stocks in the twenty-first century: implications for climate mitigation

**DOI:** 10.1007/s11027-016-9712-1

**Published:** 2016-05-13

**Authors:** Matthew Warren, Steve Frolking, Zhaohua Dai, Sofyan Kurnianto

**Affiliations:** 1 USDA Forest Service, Northern Research Station, 271 Mast Rd., Durham, NH 03824 USA; 2 Institute for the Study of Earth, Oceans, and Space, University of New Hampshire, Durham, NH USA; 3 Department of Fisheries and Wildlife, Oregon State University, Corvallis, OR USA

**Keywords:** Peatlands, Peatland rewetting, Carbon dynamics, Oil palm, Reducing emissions from deforestation and forest degradation (REDD)

## Abstract

The climate mitigation potential of tropical peatlands has gained increased attention as Southeast Asian peatlands are being deforested, drained and burned at very high rates, causing globally significant carbon dioxide (CO_2_) emissions to the atmosphere. We used a process-based dynamic tropical peatland model to explore peat carbon (C) dynamics of several management scenarios within the context of simulated twenty-first century climate change. Simulations of all scenarios with land use, including restoration, indicated net C losses over the twenty-first century ranging from 10 to 100 % of pre-disturbance values. Fire can be the dominant C-loss pathway, particularly in the drier climate scenario we tested. Simulated 100 years of oil palm (*Elaeis guineensis*) cultivation with an initial prescribed burn resulted in 2400–3000 Mg CO_2_ ha^−1^ total emissions. Simulated restoration following one 25-year oil palm rotation reduced total emissions to 440–1200 Mg CO_2_ ha^−1^, depending on climate. These results suggest that even under a very optimistic scenario of hydrological and forest restoration and the wettest climate regime, only about one third of the peat C lost to the atmosphere from 25 years of oil palm cultivation can be recovered in the following 75 years if the site is restored. Emissions from a simulated land degradation scenario were most sensitive to climate, with total emissions ranging from 230 to 10,600 Mg CO_2_ ha^−1^ over 100 years for the wettest and driest dry season scenarios, respectively. The large difference was driven by increased fire probability. Therefore, peat fire suppression is an effective management tool to maintain tropical peatland C stocks in the near term and should be a high priority for climate mitigation efforts. In total, we estimate emissions from current cleared peatlands and peatlands converted to oil palm in Southeast Asia to be 8.7 Gt CO_2_ over 100 years with a moderate twenty-first century climate. These emissions could be minimized by effective fire suppression and hydrological restoration.

## Introduction

Tropical peatlands contain a globally significant carbon (C) pool with current estimates ranging from 40 to 90 Gt C (Page et al. 2011; Yu et al. 2010). About 56 % of the world’s tropical peatland area and 77 % of the tropical peat C store occur in Southeast Asia (Page et al. 2011). Indonesia alone contains about 47 % of tropical peatlands globally, where expansive low-lying coastal plains with ample precipitation and poor drainage favor the formation of forested ombrotrophic peatlands (Dommain et al. 2014).

Peatland formation in Southeast Asia has functioned as a long-term C sink for thousands of years, with most peatlands initiating during the mid-Holocene around 6000–8000 years ago (Dommain et al. 2011). Peat swamp forest soils are waterlogged most of the year and the anaerobic, nutrient poor conditions prevent the complete decomposition of forest litter. Long-term average rates of peat accumulation are relatively slow, ranging from 0.5 to 1.8 mm year^−1^, sequestering 0.5–1.5 Mg C ha^−1^ year^−1^ (Dommain et al. 2011). However, as processes of peat formation have occurred for millennia, peat layers over 4 m thick are common and deposits over 10 m thick are reported from Riau, Sumatra, and the upper Kapuas basin in West Kalimantan, Indonesian Borneo (Brady 1997; Warren et al. 2012). The C density of tropical peat normally ranges from about 50 to 70 kg m^−3^, therefore C accumulated in peat greatly exceeds that of living forest biomass (Warren et al. 2012). Peat C stocks over 2000 Mg C ha^−1^ are common and over 7500 Mg C ha^−1^ are stored in exceptionally thick peat layers exceeding 12 m (Jaenicke et al. 2008; Murdiyarso et al. 2010; Warren et al. 2012). By comparison, the biomass of tropical rainforests typically stores between 130 and 240 Mg C ha^−1^ (IPCC 2006), equivalent to the amount of C stored in about 35 cm of peat.

In addition to their capacity to sequester and store C, tropical peat swamp forests supply numerous ecosystem services including hydrological regulation and provision of forest products. They also contain high biodiversity and are habitat for many rare and endangered species including Sumatran tigers, leopards, orangutans, and gibbons (Cheyne and Macdonald 2011; Posa et al. 2011; Nowak 2013; Wich et al. 2008). Despite these values, Southeast Asian peatlands are being deforested, drained and burned at very high rates, mostly for conversion to industrial oil palm (*Elaeis guineensis*) and pulp and paper (*Acacia* spp*.*) plantations (Langner et al. 2007; Miettinen and Liew 2010; Margono et al. 2014). For example, Miettinen et al. (2011a, b) estimated that Sumatra lost 41.3 % of its peat swamp forest cover from 2000 to 2010, and the island of Borneo lost 24.8 % over the same period. Furthermore, Margono et al. (2014) reported that from 2000 to 2012, Indonesia lost 13.2 % of its primary wetland forests, with Sumatra losing 36.1 % of its primary wetland forest cover.

The deforestation, degradation, and conversion of Southeast Asian peatlands produce net greenhouse gas emissions to the atmosphere from the decomposition and combustion of C-dense surface peat layers (Frolking et al. 2011a; Turetsky et al. 2015). Peat decomposition is estimated to release between 275 and 120 Mg CO_2_–C ha^−1^ for oil palm and *Acacia* plantations, over rotation periods of 25 and 6 years, respectively (Drösler et al. 2014). Intergovernmental Panel on Climate Change (IPCC) default emission guidelines for computing national emission inventories suggest an additional 88 Mg C ha^−1^ are released to the atmosphere from land-clearing peat fire (Drösler et al. 2014). Average total C emissions from peat forest conversion to oil palm plantation are estimated to be between 350 and 425 Mg C ha^−1^ over a 25-year crop rotation (Hergoualc’h and Verchot 2011, 2014; Kurnianto et al. 2015). Using a modeling approach, Kurnianto et al. (2015) estimated C loss of peatlands converted to oil palm plantations to be about 1400 Mg C ha^−1^ over 100 years, equivalent to 2900 years of C accumulation.

Climate mitigation via forest and land management refers to strategies aimed to conserve ecosystem C stocks and maintain or increase C sequestration. The climate mitigation potential of tropical peatlands has gained attention in recent years as large and persistent greenhouse gas emissions can be avoided or decreased if peatlands remain intact or are rehabilitated (Murdiyarso et al. 2010). In addition, peatland conservation or rehabilitation for climate mitigation also includes multiple co-benefits such as maintenance or restoration of ecosystem services, biodiversity, and air quality from reduced fire occurrence. These added benefits provide additional incentive to better manage peatland resources (Page et al. 2009). However, assessing avoided emissions, C dynamics, and co-benefits derived from climate mitigation projects on peatlands remains challenging. Inventory guidelines and methodologies have only recently become available and are based on few data from a limited number of sites (Drösler et al. 2014). Few heuristic tools are available to evaluate the impact of management practices on C dynamics in tropical peatlands and potential climate mitigation benefits of peatland restoration. Most C calculators and other tools to inventory greenhouse gas emissions from land-use change apply to non-wetland forest types, and do not account for significant C losses from peat decomposition and burning.

In this study, we used the Tropical Holocene Peat Model (HPMTrop)—a process-based dynamic tropical peatland C model (Kurnianto et al. 2015)—to simulate C dynamics of several hypothetical peatland land-use management scenarios. Furthermore, we estimate the impacts of land use and restoration on total peatland CO_2_ emissions under three simulated twenty-first century climate regimes. The modeled C losses and gains from each scenario are compared to assess the climate mitigation potential of alternative management practices. The scenarios included in the study represent climate impacts only, peat swamp forest clearing and abandonment, conversion to oil palm for multiple crop rotations, and conversion to oil palm and subsequent forest restoration.

Carbon dynamics for each scenario are modeled to the year 2100, using three climate models. Using HPMTrop to assess C dynamics under these scenarios allows us to evaluate the climate mitigation potential of various peatland restoration interventions including fire suppression, hydrological restoration, and forest restoration. The purpose of the study is to use a process-based model to broadly inform land management policy in terms of C gains and losses of tropical peatlands, particularly when restoration alternatives aimed to abate greenhouse gas emissions are being considered.

## Methods

### Tropical peat accumulation model—HPMTrop

**Fig. 1:**
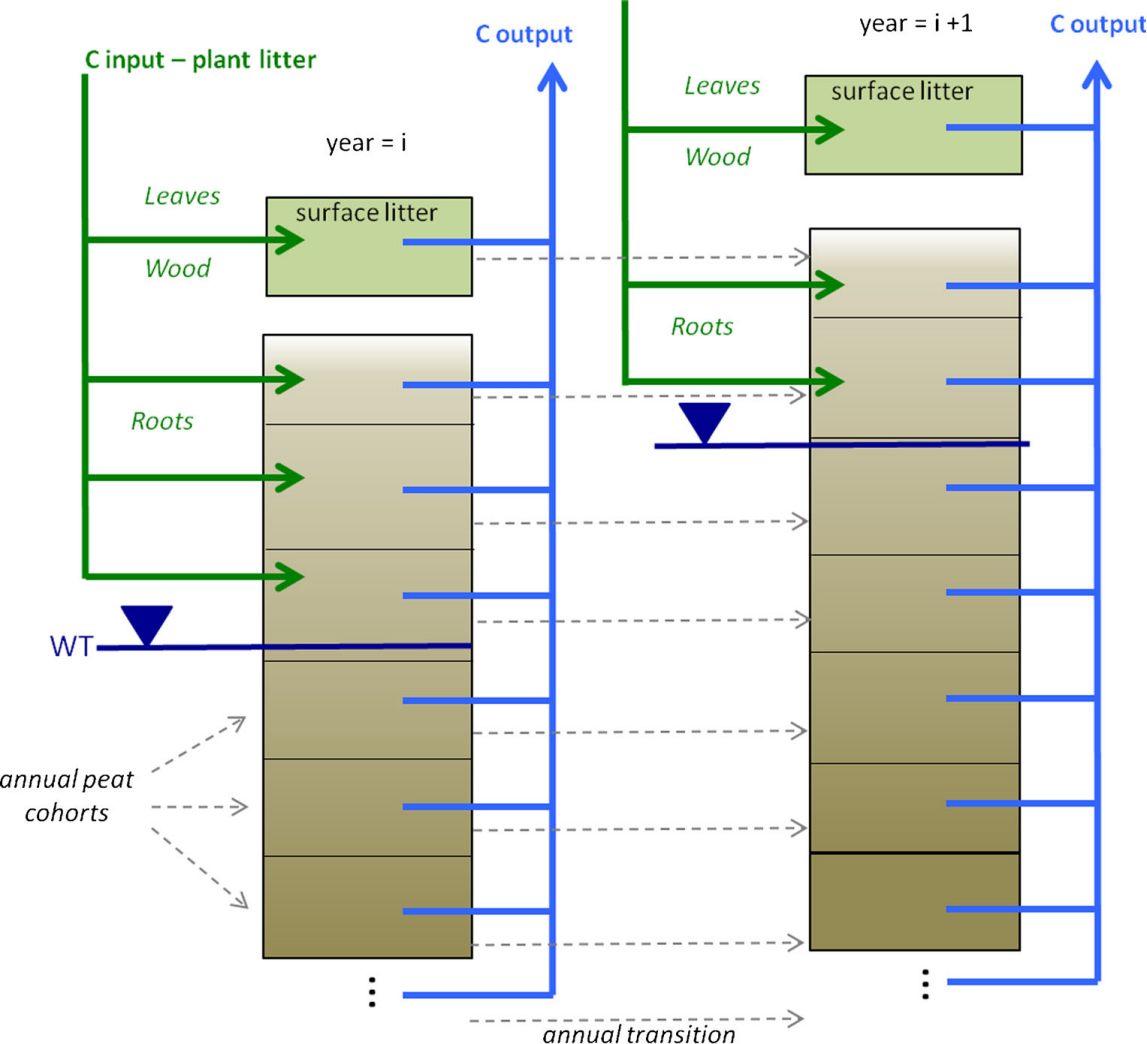
Conceptual diagram of HPMTrop model peat carbon balance. Monthly litter inputs add carbon to a surface litter pool and shallow peat, and monthly decomposition removes carbon from the surface litter and all annual peat cohorts. At the end of each simulation year, the remaining surface litter becomes the top annual peat litter cohort; through this process over millennia, a stratified column of annual peat cohorts accumulates. Water table position (*WT*) varies from month to month with precipitation deficit (see text)

HPMTrop simulates peat accumulation in tropical peat swamp forests over millennia (Kurnianto et al. 2015), through the balance of above- and below-ground litter inputs and litter/peat decomposition. It is driven by monthly precipitation and uses an empirical function relating accumulating precipitation deficit (see Sect. 2.2 below) to monthly water table depth. Annual peat cohorts are composed of annual aboveground (leaf and wood) litter that has not decomposed by the end of the year it falls, and they subsequently gain mass through root litter inputs while in the root zone, and lose mass by decomposition (Fig. 1). The model is described in greater detail in Kurnianto et al. (2015) and Frolking et al. (2010), including references for all parameter values; here we provide a summary of its basic functionality. Litter inputs for peat swamp forest are 0.079 kg m^-2^ month^−1^ for leaves, 0.057 kg m^-2^ month^−1^ for wood, and 0.025 kg m^-2^ month^−1^ for roots. Monthly litter production is simulated to be a very weak function of water table depth (Kurnianto et al. 2015). Wood and leaf litter accumulates in a litter layer, which at the end of the year becomes a surface peat layer, overlying annual peat layers generated in earlier years. Root inputs are added to peat layers in the rooting zone in proportion to layer thickness. Litter and peat decomposition rates are computed individually for each annual layer and each litter type (leaf, wood, root) using litterbag study values for initial rates (*k*_0_; 0.106, 0.022, and 0.069/month for leaf, wood, root, respectively), with the rates subsequently declining with mass lost (*k(t)* = *k*_0_·[*m(t)*/*m*_0_], where *m*_0_ is the total litter input to the layer, and *m(t)* is the mass remaining at time *t*; (Clymo et al. 1998; Frolking et al. 2002). Decomposition rates are also modified by position relative to the monthly water table, which controls unsaturated zone water content, and is a proxy for oxygen penetration into the saturated zone (Frolking et al. 2010). For oil palm vegetation, litter input rates are 0.025 kg m^-2^ month^−1^ for leaves, 0.06 kg m^-2^ month^−1^ for roots, and zero for wood, and initial leaf and root litter decomposition rates are 0.09 month^−1^ (Kurnianto et al. 2015).

Peat accumulation rates in tropical peat swamp forests are highly variable, ranging from ~0.2 to ~2 mm year^−1^ for individual sites (Dommain et al. 2011). The initial HPMTrop model study simulated peat height and C accumulation rates for inland and coastal peat swamp forests in Indonesia as ~0.5 and ~1.1 mm year^−1^ (respectively). These rates are similar to mean long-term peat accumulation rates determined by radiocarbon dating of several peat cores in Southeast Asia (Dommain et al. 2011; Kurnianto et al. 2015). The initial Kurnianto et al. (2015) model study also reported conversion of peat swamp forest to oil palm production for 100 years caused a loss of 1200–1400 Mg C ha^−1^ over 100 years, with ~60 % of the loss from lower litter C inputs and enhanced decomposition due to draining, and ~40 % from a prescribed fire at the beginning of each 25-year oil palm rotation. These losses were equivalent to the past ~3000 or ~6000 years of peat accumulation in the coastal and inland peatlands, respectively. It is important to note that in the Kurnianto et al. (2015) study and in the simulation results presented below, HPMTrop only tracks peat C, so results do not include ecosystem losses of forest biomass C due to clearing, nor C accumulations due to recovery of forest biomass.

For this study, we made several modifications to HPMTrop. First, the model now uses both the stochastic monthly precipitation for 5000 to 0 BP (Kurnianto et al. 2015) and historical (1950 to 2005 CE) and future (2006-2099 CE) monthly precipitation from climate models (see Sect. 2.2 below) to simulate the peat C balance from initiation at 5000 BP through land use and climate change impacts in the twenty-first century. Second, we added functionality for a linear increase in litter input from zero to full pristine peat swamp forest rates over 25 years (leaf, wood, and root as above) for the forest restoration scenarios (optimal and sub-optimal). We assume that litter input reaches pristine forest levels faster than total forest biomass. Third, we added functionality to decrease the effectiveness of drainage ditches over time, if the peatland is losing peat and the ditch is not maintained (we assume ditches are maintained while oil palm is cultivated). The distance that the ditch lowers the water table declines proportional to peat height loss, implying that the bottom of the ditch is at a fixed height relative to the base of the peat profile, and the peat surface lowers relative to the bottom of the ditch due to losses from burning or net decomposition. Note that HPMTrop does not simulate physical compaction of peat with drainage. Finally, we added stochastic fire functionality, used in two of the scenarios, whereby probability of fire is a function of how dry the weather is, and the thickness of the peat layer burned is a function of water table depth. Simulated fires remove all peat down to water-table-dependent burn depth, have no impact on the deeper peat, and the model does not include the small amount of pyrogenic materials (charcoal, soot) that would be resistant to further decomposition.

### Climate drivers

All simulations were run from coastal peat initiation (set at 5000 BP; Dommain et al. 2014) to 2099 CE. HPMTrop requires monthly precipitation as a driver. For 5000 to 0 BP (=1950 CE), we used the stochastic precipitation reconstruction algorithm developed by Kurnianto et al. (2015). For 1950 to 2099 CE, we used three climate model simulations—historical for 1950–2005 CE, and projected for 2006–2099 CE—taken from the bias-corrected output from the Inter-Sectoral Impact Model Intercomparison Project (ISI-MIP; Warzawski et al. 2014). ISI-MIP bias-corrected precipitation output from five climate models—Geophysical Fluid Dynamics Laboratory Earth System Model version 2M (GFDL-ESM2M; referred to here as GFDL), Hadley Centre Global Environment Model version 2ES (HadGEM2-ES), Institut Pierre Simon Laplace Climate Model version 5A-Low Resolution (IPSL-CM5A-LR), Model for Interdisciplinary Research on Climate, Earth System Model with coupled Atmospheric Chemistry (MIROC-ESM-CHEM; referred to here as MIROC), and Norwegian Earth System Model version 1-M (NorESM1-M; referred to here as NorESM)—to the Water and Global Change (WATCH; http://www.eu-watch.org/) historical precipitation reconstruction (Weedon et al. 2011).

**Fig. 2:**
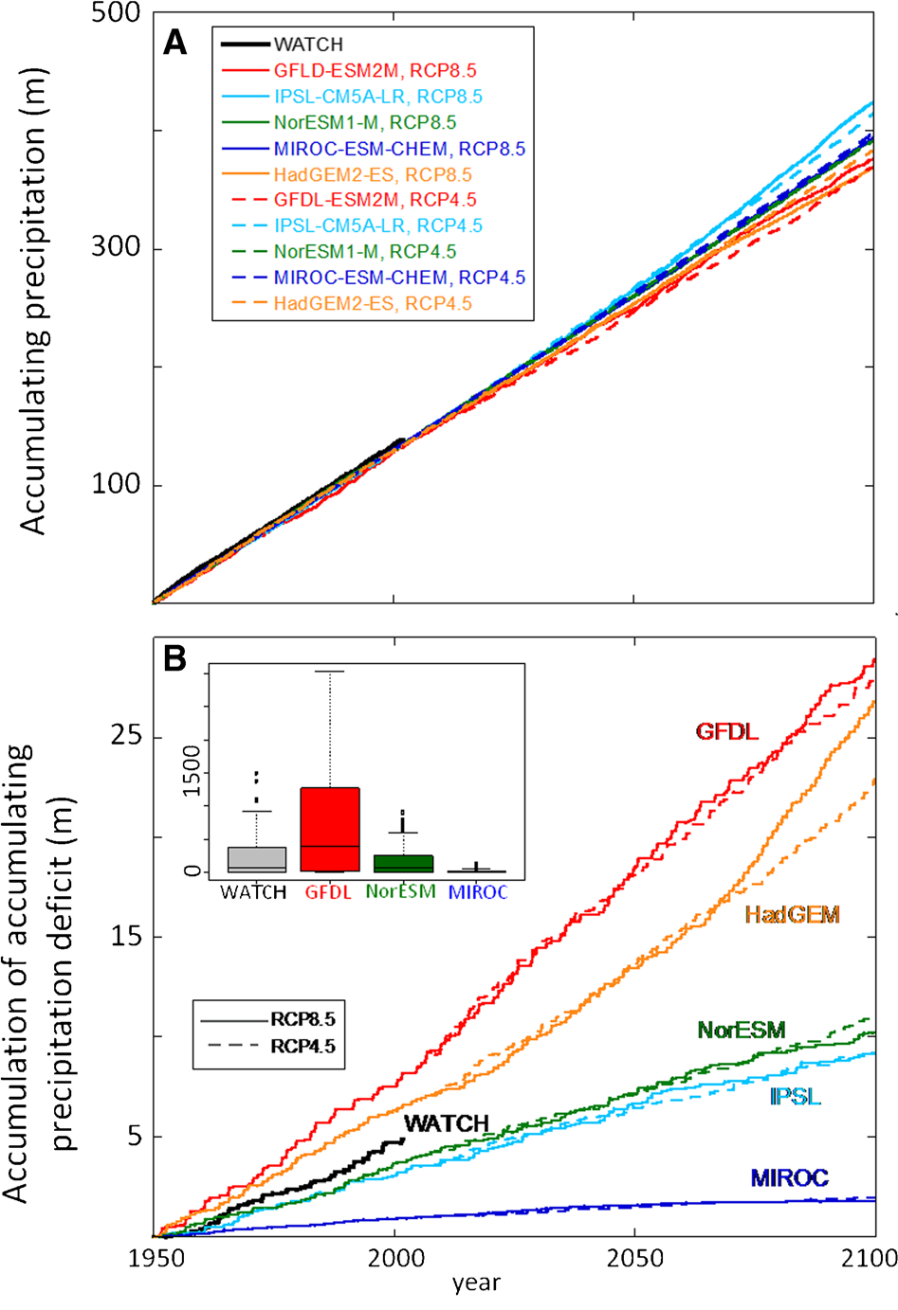
**a** Accumulating precipitation for WATCH reconstruction (*black line*; 1950–2005) and ten climate model simulations (1950–2100)—five models (*colored lines*) for both RCP4.5 (moderate emissions; *dashed lines*) and RCP8.5 (high emissions; *solid lines*) simulations—which were bias corrected to the WATCH historical data (Weedon et al. 2011; Hempel et al. 2013). Model annual precipitation is consistent with WATCH to 2005 and generally continues at similar annual rate through 2100. **b** Accumulation of annual cumulative precipitation deficit (see text) for WATCH and the same ten climate model simulations. Models have very different dry season precipitation, ranging from much drier than WATCH historical (*GFDL*) to much wetter than WATCH historical (*MIROC*). Note that the cumulative precipitation deficit (see text) is reset to zero during the wet season each year, so while (**b**) conveys a difference between the models, it does not represent an actual accumulating soil water deficit. **b** *Inset*, box plot of range of annual cumulative (sum of monthly) water deficit values, in millimeters (WATCH, 1950–2001; climate models, 1950–2099)

We selected a grid cell (2.75° S, 113.75° E center) in coastal Central Kalimantan, Indonesia, as a representative peatland location. For this location, the five models had bias-corrected annual precipitation consistent with WATCH for 1950-2005, and all continued to 2100 with fairly constant and similar annual precipitation (Fig. 2a). We also computed a monthly accumulating precipitation deficit (Malhi et al. 2009; Frolking et al. 2011b), *P*_i_^*^ (mm), as

1 $$ {P}_i^{*}= \max \left[0,\kern0.5em {P}_{i-1}^{*}+\left(100-{P}_i\right)\right] $$

where *i* refers to the month, *P*_*i*_ is monthly precipitation (mm), and the max[ ] function selects the maximum of the two arguments (so *P*_i_^*^ ≥ 0). This precipitation deficit is a measure of dry season length and aridity, and in the humid tropics precipitation is sufficient to reset *P*_i_^*^ to zero every wet season. *P*_i_^*^ is used by HPMTrop to empirically compute the peat swamp forest monthly water table depth (WTD_*i*_ = 2*P*_i_^*^; Kurnianto et al. 2015).

Although total annual precipitation was not significantly different among models, the five climate models had very different dry season precipitation, as quantified by *P*_i_^*^, during both the historical and twenty-first century periods, with the GFDL model being the driest, MIROC the wettest, and NorESM intermediate and similar to WATCH for 1950-2005 (Fig. 2b). Model annual precipitation and monthly accumulating precipitation deficit were generally similar for both Representative Concentration Pathway (RCP)4.5 (moderate emissions) and RCP8.5 (high emissions) climate scenarios (Fig. 2). We selected the GFDL, MIROC, and NorESM RCP8.5 precipitation scenarios to drive HPMTrop simulations from 1950 to 2099 CE, to provide a wide range of future precipitation scenarios. We refer to these as the drier (GFDL), moderate (NorESM), and wetter (MIROC) climate scenarios.

### Land-use change scenarios

We evaluated peat C dynamics of six land-use scenarios using HPMTrop (Table 1). These scenarios are designed to capture a range of possible land-use impacts on peat C stocks and are meant to be only a general representation of many possibilities. There is substantial uncertainty in many aspects of all scenarios—future climate, changes in precipitation seasonality, fire occurrence probabilities, and disturbed peatland restoration trajectories. In developing the scenarios, we have combined some simple bounding assumptions in order to evaluate their relative impacts—wetter, moderate or drier climate, higher, lower, or zero fire occurrence probability, and rapid, slow, or no restoration. We consider only oil palm production as a land-use change; industrial pulp plantations and other common land-use conversions requiring drainage lose peat C at similar rates; however, field data are lacking to parameterize HPMTrop to simulate C dynamics in these systems (Murdiyarso et al. 2010).

**Table 1:** Land-use and restoration scenarios

Scenario	5000 BP–0 BP	1951–1999	2000–2024	2025 – 2099
S1. Pristine forest remaining forest; no land use or land cover change	V: forest; P: stochastic; W: 0.0 m; F: none	V: forest; P: CMs; W: 0.0 m; F: none	V: forest; P: CMs; W: 0.0 m; F: none	V: forest; P: CMs; W: 0.0 m; F: none
S2. Peatland degradation; forest cleared, burned, and abandoned, minimal draining not maintained, high probability of fire.	Same as above	Same as above	V: ramp to forest^a^; P: CMs; W: 0.3 m, declining^b^; F: high-frequency stochastic	V: increase to forest^a^; P: CMs; W: 0.3 m, declining^b^; F: high-frequency stochastic
S3. Forest conversion to oil palm; forest cleared, burned and drained. Four 25-year oil palm rotations with prescribed fires.	Same as above	Same as above	V: oil palm (1×); P: CMs; W: 0.6 m; F: OP rotation^c^	V: oil palm (3×); P: CMs; W: 0.6 m; F: OP rotations^c^
S4. Forest conversion to oil palm; forest cleared, burned and drained. Four 25-year oil palm rotations with one initial prescribed fire.	Same as above	Same as above	Same as above	V: oil palm (3×); P: CMs; W: 0.6 m; F: none
S5. Forest conversion to oil palm; forest cleared, burned, drained. One 25-year oil palm rotation followed by “optimum” restoration: forest recovers, drainage blocked, and no fire.	Same as above	Same as above	Same as above	V: increase to forest^a^; P: CMs; W: 0.0 m; F: none
S6. Forest conversion to oil palm (as no. 4); followed by abandonment. Forest sub-optimal recovery, drainage not maintained, low probability of fire.	Same as above	Same as above	Same as above	V: increase to forest^a^; P: CMs; W: 0.6 m, declining^b^; F: low-frequency stochastic

Note that 0 BP = 1950 CE

*V* vegetation, *P* precipitation (climate models (CMs)), *W* water table depth offset (down from peat surface) relative to pristine forest monthly water table depth simulation and fire (F)

^a^Vegetation litter input to peat ramps from zero to full forest input in 25 years; restarts at zero following fire

^b^Ditch effectiveness at lowering water not maintained; effectiveness declines linearly with peat height lost (see text)

^c^ Fire at onset of each 25-year oil palm rotation burns 20 cm of peat

All scenario results include only changes in peat C, ignoring the initial forest biomass that is cleared and removed from the site (harvest or burning) in scenarios 2–6, forest biomass recovery in scenarios 2, 5, and 6, and oil palm vegetation biomass in scenarios 3–6. Two scenarios—a control scenario of peat forest remaining as forest (S1), and conversion to 25-year oil palm rotations for 100 years with prescribed fire between rotations (S3)—follow Kurnianto et al. (2015), except that the scenarios now include impacts of twenty-first century climate change from three models (described above), while Kurnianto et al. (2015) used only the stochastic Holocene climate reconstruction. The other four scenarios expand the analysis done by Kurnianto et al. (2015), modifying land use to include two restoration scenarios, optimal (S5) and sub-optimal (S6), and degradation (clearing followed by abandonment, S2). The degradation and sub-optimal restoration scenarios (S2 and S6, respectively) include stochastic fire occurrence with corresponding higher and lower fire frequencies. Fire frequency and depth of peat burned increase with drought severity, represented here by increasing monthly accumulating precipitation deficit (Putra et al. 2008; Field and Shen 2008). We did not find sufficient published data to develop an empirical fire probability model for disturbed tropical peatlands, so we developed two general ad hoc parameterizations: lower probability for the sub-optimal recovery following oil palm scenario (S6), with fire return intervals (based on 10^4^ fire-probability realizations with each climate model) of about 300, 125, and 70 years in the wet, moderate, and dry climates, respectively, and higher probability for the degradation scenario (S2), with fire return intervals of about 20, 10, and 5 years for the wet, moderate, and dry climates, respectively. Given the lack of an empirical model, these probabilities met our intended goal of a scenario (S6) in which the majority of the simulations had no fires, and a scenario (S2) in which the simulations had multiple fires (see Fig. 4). Stochastic fires burn up to 20 cm (S2) or 15 cm (S6) of surface peat, with burn depth linearly increasing with the magnitude of the monthly accumulating precipitation deficit. For simulations with the wetter climate, fires are infrequent and do not consume much peat, while for simulations with the drier climate fires are more common and tend to burn 10–20 cm of peat (Agus et al. 2013).

Scenario 1 is a pristine coastal peat swamp forest, with no land-use change or restoration. Therefore, scenario 1 represents natural peatland C dynamics under the three models of future climate. Scenario 2 is a land degradation scenario (Blackham et al. 2014) where the pristine forest is cleared and removed in 2000 CE. Moderate drainage is assumed, lowering the water table 0.3 m below pristine monthly water table depth, and there is a high probability of fire occurrence with a return frequency of about 10 years in the moderate climate, and fire probability and burn depth proportional to monthly accumulating precipitation deficit. Vegetation litter input to the peat increases linearly from zero towards full pristine peat swamp forest litter input (see Sect. 2.1 above) over 25 years, but rarely reaches that point because of frequent resetting to zero by fire. Due to the stochastic fire probability in S2, we ran HPMTrop 25 times with each climate scenario to generate a distribution of simulation results. The land degradation scenario is designed to be a general representation of the 2.3 million ha of Southeast Asian peatlands that have been cleared and minimally drained, but are not immediately planted and remain in a degraded state where secondary forest succession is inhibited by fire (Page et al. 2009, Koh et al. 2011).

Scenarios 3 to 6 include land-use change initiating in 2000 CE, with or without subsequent restoration. Scenarios 3 and 4 represent pristine coastal peat swamp forest converted to oil palm plantation in 2000 CE, with four 25-year oil palm rotations during the twenty-first century. Oil palm land-use impacts follow those simulated in Kurnianto et al. (2015), with ditching to lower the water table 0.6 m below pristine monthly water table depth (Agus et al. 2013), an initial fire that removes all standing forest biomass and 20 cm of peat, and oil palm litter inputs into the peat at 53 % of pristine litter inputs (only 26 % when weighted by initial decomposability) (Kurnianto et al. 2015). The two scenarios differ in that scenario 3 has a fire removing the top 20 cm of peat at the beginning of each oil palm rotation, while scenario 4 has a fire only at the beginning of the first oil palm rotation, representing improved management practices to reduce burning on peatlands.

Scenarios 5 and 6 include conversion of pristine coastal peat swamp forest to a single 25-year oil palm rotation starting in 2000 CE, with initial burning as in scenarios 3 and 4. In 2025 CE, scenario 5 undergoes optimal restoration and scenario 6 undergoes abandonment with some fire suppression, representing sub-optimal restoration. Optimal restoration (S5) means (i) immediate blockage of drainage ditches and full rewetting, (ii) no subsequent fires, and (iii) litter inputs increase linearly from zero to full pristine peat swamp forest values over 25 years. Sub-optimal restoration (S6) has (i) no blockage of drainage ditches, though the depth of the ditches declines over time according to peat height loss from decomposition and burning; (ii) stochastic fires with a return frequency of about 100 years in the moderate climate, and fire probability and burn depth proportional to monthly accumulating precipitation deficit; and (iii) litter inputs increasing linearly from zero to full pristine peat swamp forest values over 25 years, which are reset to zero at each fire. Due to the stochastic fire probability in S6, we ran HPMTrop 25 times with each climate scenario to generate a distribution of simulation results.

## Results

**Fig. 3:**
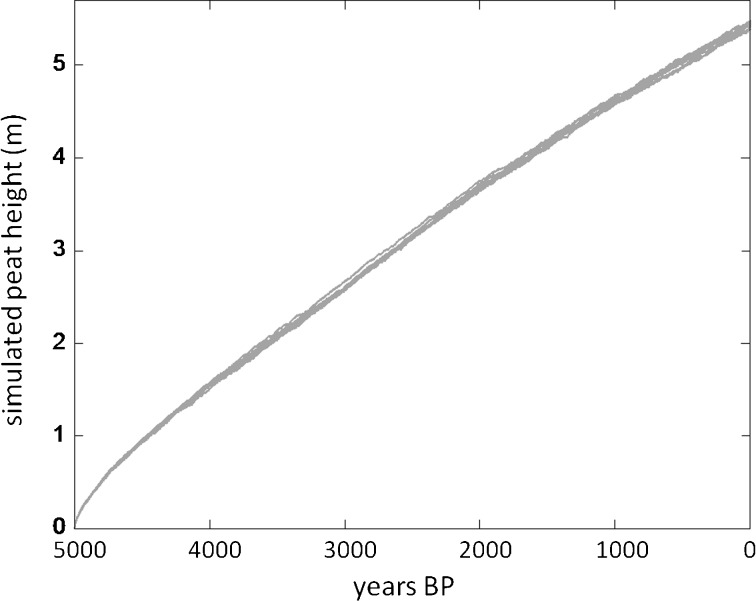
Simulated peat height for initial 5000 years of twelve simulations, 5000 BP to 0 BP (or to 1950 CE), before onset of land-use scenarios in the simulations or climate model precipitation inputs. Variation is due to stochastic precipitation reconstruction (see Kurnianto et al. 2015)

**Fig. 4:**
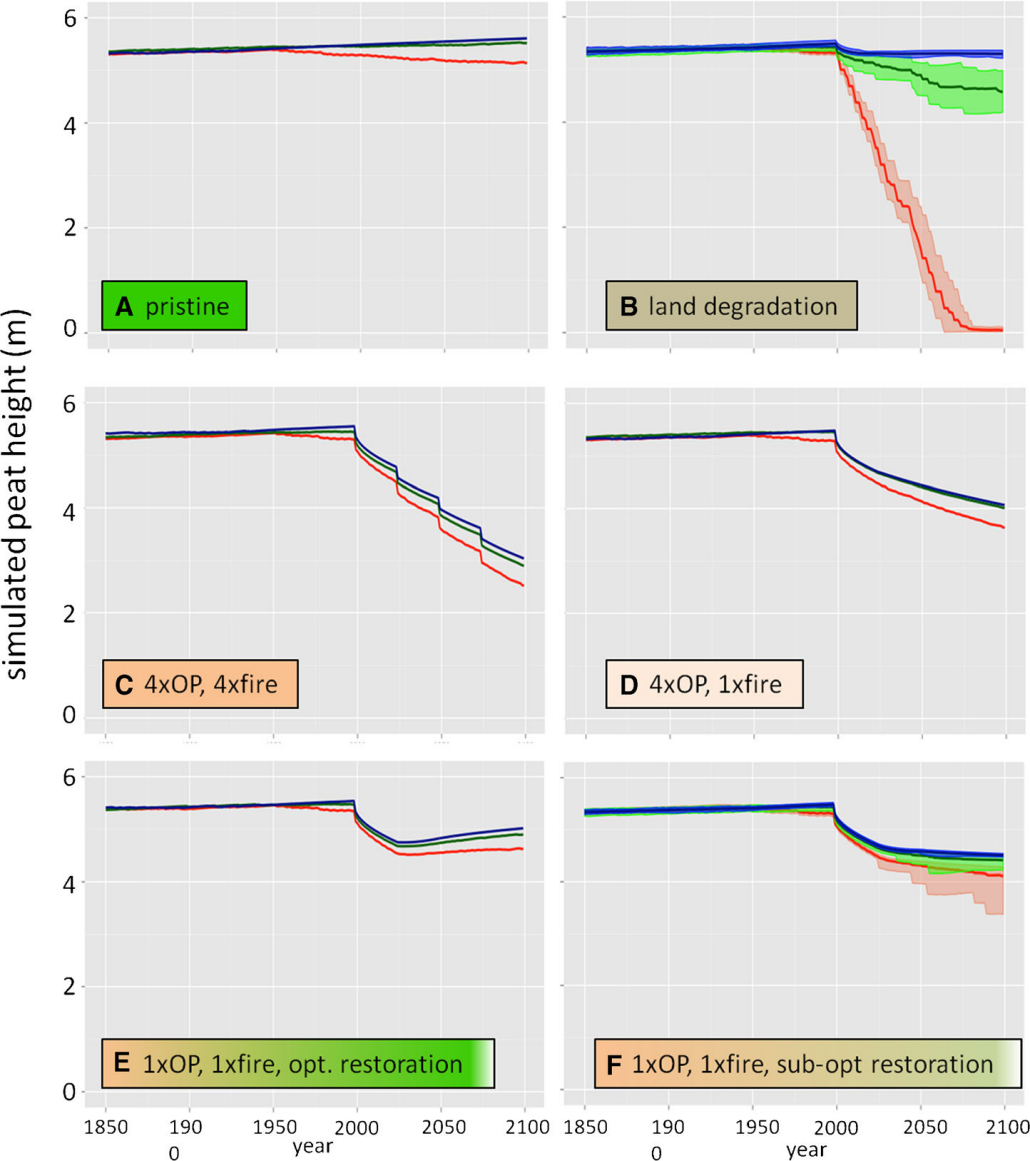
Simulated peat height for final 250 years of simulation (1850–2100 CE) for land-use scenarios. **a** S1, pristine forest; **b** S2, land degradation; **c** S3, four-rotation oil palm with fires; **d** S4, four-rotation oil palm with one fire; **e** S5, one-rotation oil palm with fire and optimal restoration; and **f** S6, four-rotation oil palm with fire and sub-optimal restoration, for three climate models—GFDL (*red*), HadESM (*green*), and MIROC (*blue*). In (**b**) and (**f**), *light lines* are 25 simulations with stochastic fire occurrence, and *heavy colored lines* are means of the 25 simulations. Climate model scenarios begin in 1950 (bias-corrected historical 1950–2005) and continue to 2100. See Table 1 for land-use scenario descriptions

All scenarios began with 5000 years of stochastic precipitation reflecting the Holocene climate, as in Kurnianto et al. (2015), and accumulated about 5.4 m or 2900 Mg C ha^−1^ of peat (1.1 mm year^−1^ or 0.58 Mg C ha^−1^ year^−1^), with small variation (<1 %) due to stochastic climate (Fig. 3). In 1950 CE (or 0 BP), the simulations all switched to precipitation drivers from historical climate model simulations (Fig. 2); for the two climate models with moderate and wetter dry seasons (NorESM and MIROC; Fig. 2b), peat accumulation continued on the Holocene trajectory in the pristine forest scenario (S1), and until land-use change in 2000 for the other five scenarios (Fig. 4). However, the drier dry season in the GFDL model caused a slow natural peat loss, as a lower water table enhanced decomposition to rates exceeding litter inputs in most years (Fig. 4a). There was a loss of about 130 Mg C ha^−1^ over 150 years (Table 2), equivalent to about 4.5 % of the peat C mass in 1950, or a decline in peat depth of about 1.6 mm year^−1^.

**Table 2:** Peat height (m) at time of maximum value and at end of simulation, simulated peat carbon mass (Mg C ha^−1^) in 1950 CE (onset of historical climate forcing in all scenarios), change in peat carbon mass from 1950 to 1999 (onset of land-use changes in scenarios 2–6), 2000–2024 (first oil palm rotation in scenarios 3–6, and onset restoration/abandonment in scenarios 5 and 6), 2025–2099 (remainder of simulation), and 1950–2099

Scenario	Height (m)		Mass	Δ-mass	EF	Δ-mass	EF	Δ-mass	EF	Δ-mass	Total Emissions^f^
	Max	Final	1950^a^	1950–1999^b^		2000–2024^c^		2025–2099^d^		Total (1950–2099)	
GFDL											
1. Pristine forest	5.4	5.1	2900	−53	4.0	−30	4.4	−49	2.4	−130	480
2. Land degradation	5.4	<0.1	2900	−52	3.9	−970	140	−1900	93	−2900	10,600
3. 4× oil palm with 4 fires	5.4	2.5	2900	−60	4.5	−330	48	−950	47	−1300	4800
4. 4× oil palm with 1 fire	5.4	3.6	2900	−53	4.0	−320	47	−450	22	−820	3000
5. 1× oil palm, restoration	5.5	4.6	2900	−54	4.0	−320	47	+46	−2.3	−330	1200
6. 1× oil palm, abandonment	5.4	4.1	2900	−52	3.9	−330	48	−210	10	−590	2200
NorESM											
1. Pristine forest	5.5	5.5	2900	+7.8	−0.6	+6.4	−0.9	+27	−1.3	+41	−150
2. Land degradation	5.4	4.6	2900	+8.8	−0.7	−180	26	−270	13	−440	1600
3. 4× oil palm with 4 fires	5.4	2.9	2900	+8.1	−0.6	−300	44	−850	42	−1200	4400
4. 4× oil palm with 1 fire	5.5	4.0	2900	+7.8	−0.6	−300	44	−360	18	−650	2400
5. 1× oil palm, restoration	5.5	4.9	2900	+6.5	−0.5	−310	46	+110	−5.4	−194	710
6. 1× oil palm, abandonment	5.4	4.4	2900	+7.8	−0.6	−300	44	−130	6.4	−420	1500
MIROC											
1. Pristine forest	5.6	5.6	2900	+35	−2.6	+16	−2.3	+50	−2.4	+100	−370
2. Land degradation	5.5	5.3	2900	+38	−2.8	−97	14	−2.7	0.1	−62	230
3. 4× oil palm with 4 fires	5.5	3.0	3000	+36	−2.7	−300	44	−830	41	−1100	4000
4. 4× oil palm with 1 fire	5.5	4.1	2900	+35	−2.6	−300	44	−340	17	−610	2200
5. 1× oil palm, restoration	5.5	5.0	2900	+40	−3.0	−300	44	+140	−6.9	−120	440
6. 1× oil palm, abandonment	5.5	4.5	2900	+39	−2.9	−300	44	−110	5.4	−370	1400

Emission factors (EF; Mg CO_2_ ha^−1^ year^−1^) are given for each time interval; negative values indicate carbon sequestration. Total emissions over the 100-year simulation are provided in Mg CO_2_ ha^−1^. Values reported for three climate models and six scenarios (means of 25 simulations for degradation restoration and abandonment scenarios 2 and 6). All values reported in two significant figures. See Table 1 for scenario details

^a^At onset of switch from Holocene climate reconstruction to climate model historical climate

^b^From switch to climate model historical climate to prior to first oil palm rotation and fire (scenarios 3–6)

^c^During 1st oil palm rotation (scenarios 3–6)

^e^End of simulation

^f^Total net emissions from 1950 to 2099 in megagrams CO_2_ per hectare

The peatland degradation scenario with clearing and shallow ditching followed by abandonment, was most sensitive to modeled future climate and lost all peat in the drier climate scenario due to high fire frequency (Fig. 4b). The resultant emissions are about 10,600 Mg CO_2_ ha^−1^ over the 100 years of simulation. In the wet climate with low fire probability and few fires, the peat height and C balance were stable after an initial C loss due to draining and low C inputs following clearing, while the moderate climate generated intermediate results (Fig. 4b). Total emissions in moderate and wet climates were 1600 and 230 Mg CO_2_ ha^−1^, respectively.

Simulated conversion to oil palm, with clearing, initial burning and deep ditching, led to a loss of about 0.6 m or 300 Mg C ha^−1^ of peat in the first 25-year rotation (Fig. 4c–f; Table 2). These results are consistent with those of Kurnianto et al. (2015). Three subsequent 25-year oil palm rotations with burning caused a loss of an additional 1.6 m or 800–900 Mg C ha^−1^ of peat (Fig. 4c; Table 2), while eliminating burning from these rotations reduced the 75-year loss to about 0.6 m or 350–450 Mg C ha^−1^ of peat, roughly the amount lost in the first 25 years with burning (Fig. 4d; Table 2). Continued losses in the oil palm scenario without fire were due to maintenance of deep drainage ditches and lower litter inputs from oil palm than the pristine forest. Climate sensitivity was only significant for the drier climate (Fig. 4c, d; Table 2), which lost about 35 % more C due to enhanced decomposition relative to the wetter climate scenarios. Total emissions from simulated 100 years of oil palm cultivation with prescribed burns between rotations were 4800, 4400, and 4000 Mg CO_2_ ha^−1^ for dry, moderate, and wet climates, respectively. Eliminating prescribed burns between each rotation reduced total emissions to 3000, 2400, and 2200 Mg CO_2_ ha^−1^, for the dry, moderate, and wet climates, respectively.

**Fig. 5:**
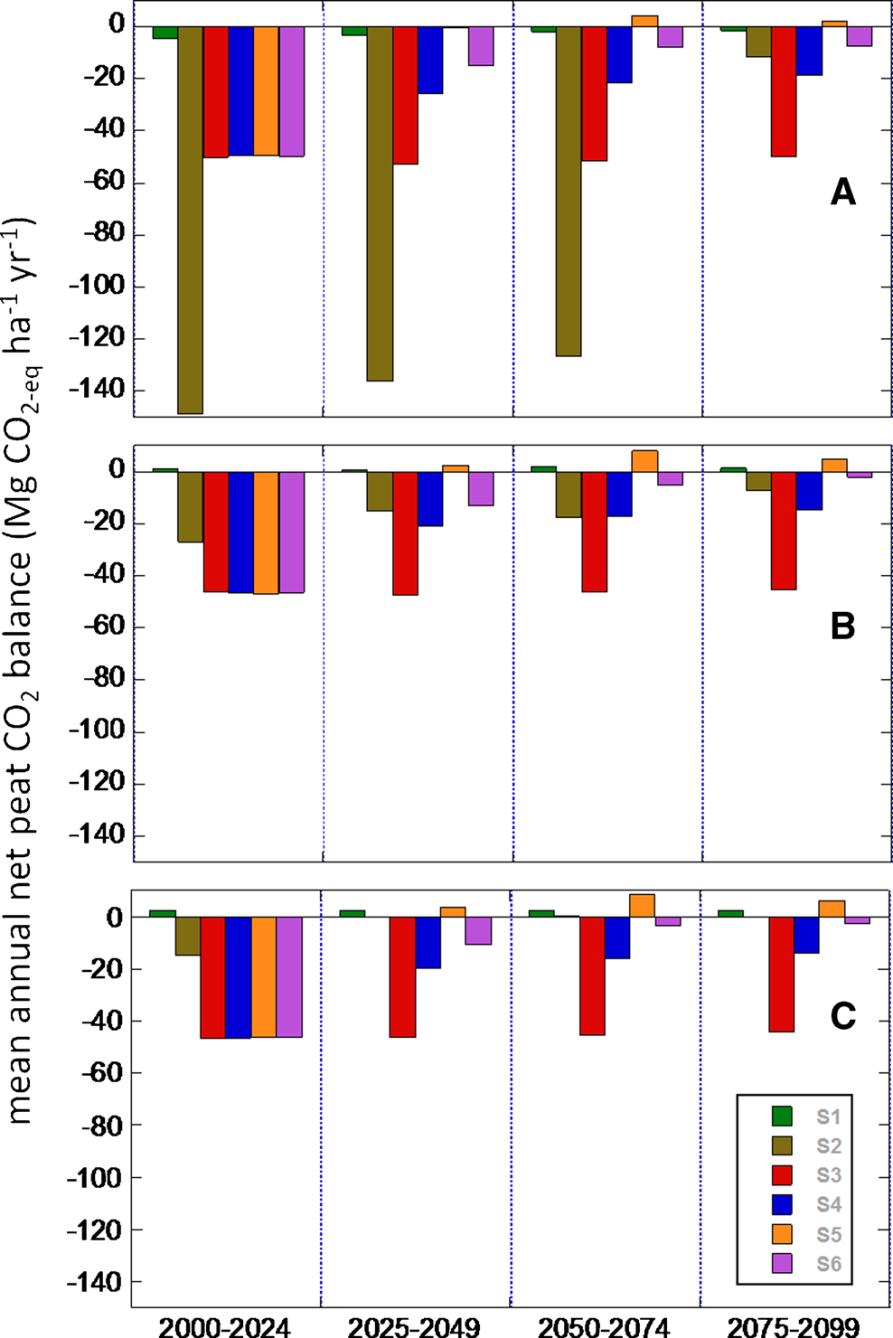
Simulated mean annual net CO_2_ balance of peat for each quarter of the twenty-first century for the six scenarios (see Table 1) and the **a** drier (GFDL), **b** moderate (NorESM), and **c** wetter (MIROC) RCP8.5 climate scenarios (see Fig. 1). Negative values refer to net CO_2_ loss from peat to the atmosphere; positive values refer to net atmospheric CO_2_ sequestration in peat

Optimal peat swamp forest restoration, including blocked drainage and full recovery of forest litter inputs over 25 years, caused the simulated peatland to resume peat accumulation at rates of about 3.3 mm year^−1^ or about 1.7 Mg C ha^−1^ year^−1^ in the wet and intermediate climate scenarios, and about 1.3 mm year^−1^ or 0.6 Mg C ha^−1^ year^−1^ in the drier scenario (Fig. 4e; Table 2). These rates are about 1.2–3 times higher than the long-term Holocene accumulation rates, because prior to restoration, the shallower, relatively labile surface peat had been burned off or oxidized during the 25-year oil palm rotation, leading to lower overall decomposition during the subsequent 75 years. Uptake rates in the restoration scenario were highest after simulated forest litter production had fully recovered (set to 25 years) and then declined as this litter production stabilized and decomposition rates slowly increased as less-decomposed peat accumulated at the surface (Fig. 5). Peat accumulation rates would be expected to return to long-term means over a few hundred years if climate remained stable. Abandonment following one 25-year oil palm rotation (scenario 6) led to slow peat loss in the wet and intermediate climate scenarios due to persistence of the ditch impacts, while peat loss continued at a more rapid rate in the drier climate scenario due to a moderate probability of fire (Figs. 4f and 5; Table 2) with some stochastic fire realizations leading to large losses (Fig. 4d–f).

The simulated peat C balance in the twenty-first century was strongly dependent on land use, and more weakly dependent on climate, except for the strong impact of a drier and/or longer dry season on simulated fire frequency (Fig. 5). The magnitude of annual, land-use generated C losses was generally much larger than pristine forest uptake rates, and also larger than the more rapid uptake rates in the optimal recovery scenario (Fig. 5).

**Fig. 6:**
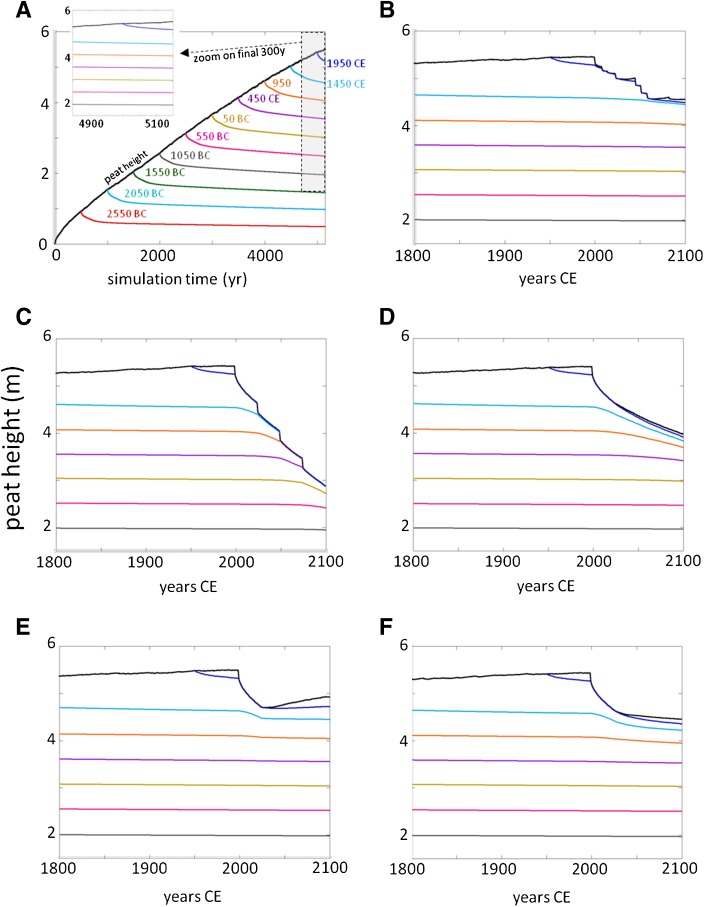
**a** Simulated peat height (*black line*) for scenario 1 (pristine PSF) and the NorESM RCP8.5 future climate, and location in peat profile over time of peat annual cohorts formed every 500 years (*colored lines*, labeled by year of formation at peat surface). Steeper declines near surface are due to rapid initial decomposition of shallow peat; slower declines at depth reflect very slow decomposition once peat is about 0.7 m below surface. *Inset*, zoom of final 300 years of simulation, 1800–2100 CE (*shaded*). **b**–**f** Final 300-year zoom (*shaded region* in (**a**)) for land-use scenarios **b** S2, **c** S3, **d** S4, **e** S5, and **f** S6; see Table 1 for scenario descriptions. Changes in slope of trajectories indicate land-use impacts below that depth in the peat; if trajectories are not perturbed, then peat below that depth is isolfated from impacts of land use. In most scenarios with the NorESM climate, significant impact is only in the upper 1–2 m of peat, except S3 with four oil palm rotations with burning in each rotation. Note that HPMTrop does not simulate drainage-induced peat compaction, so declines in peat height are due to decomposition and burning only

Finally, HPMTrop simulations show that peat lost in almost all scenarios comes from the top 1–2 m of the profile (Fig. 6). In the wet season, water tables in pristine peatland are generally at or very near the surface, so in a ditched (drained) peatland, the wet-season water table will not be deeper than the depth of the ditch (0.6 or 0.3 m in our scenarios; Table 1). Water tables can drop tens of centimeters below that in the dry season but will generally not be much deeper than about 1 m. Increased peat decomposition occurs in this drained surface peat, but the impact does not reach much deeper. Peatland fires burn surface peat, usually tens of centimeters down, below which unsaturated peat can be quite wet, even if the water table is deeper. So all land-use impacts are relatively shallow, and only progress into deeper peat if decomposition and burning remove peat from the continually lowering surface (e.g., Fig. 6b, c) and/or ditch depth is maintained as the peat surface drops (e.g., Fig. 6c, d).

## Discussion

### Impacts of future climate on peatland carbon stocks

The HPMTrop stochastic Holocene climate reconstructions drove peat accumulation from 5000 BP to 1950 CE resulting in final peat thicknesses ranging from 5.4 to 5.6 m, storing an estimated 2900 Mg C ha^−1^ in peat for all scenarios. This is in the middle range of coastal peat swamp forest peat accumulations reported by Dommain et al. (2011). By design, the simulated peatland scenarios were almost identical when climate drivers switched from the Holocene reconstruction to the climate model historical output for the GFDL (dry), NorESM (moderate), and MIROC (wet) climate regimes in 1950. The impacts of dry season variability and accumulated precipitation deficit on peat C balance were evident over the 50-year period from 1950 to 1999 under the different climate models. Prior to simulated land conversion in 2000, all scenarios lost C under the drier dry season conditions of the GFDL model. Since climate models were not significantly different in terms of total annual precipitation (Fig. 2a), it was the intensified dry season of the GFDL model (Fig. 2b) that caused the peat C balance to naturally switch from C sink to C source, losing 1.0–1.2 Mg C ha^−1^ year^−1^. Considering the uncertainty of future precipitation patterns driven by global climate change in Southeast Asia (e.g., Fig. 2), these results indicate that dry season intensification could produce a weak positive feedback of additional climate forcing from the natural release of CO_2_ from decomposing peat. Although the model does not account for possible enhanced forest productivity induced by increased soil oxygen content and nutrient mineralization or atmospheric CO_2_ fertilization effects, the increased likelihood of more frequent and severe wildfires under drier conditions (which are not included in the model simulations from 1950 to 1999) in addition to enhanced peat decomposition would likely cause net C release from undisturbed peatlands if dry seasons intensify in the future. The moderate NorESM climate model produced peat accumulation during 1950–2000 ranging from 0.13 to 0.18 Mg C ha^−1^ year^−1^, which is well below the 5000-year average peat accumulation rate of 0.58 Mg C ha^−1^ year^−1^ under HPMTrop stochastic climate reconstruction. Peat accumulation rates under the MIROC wet climate regime were slightly above the historical average, ranging from 0.7 to 0.8 Mg C ha^−1^ year^−1^.

Peatlands can only sequester C slowly, so their large C stocks result from millennia of slow peat accumulation with very low disturbance rates. Overall, our results indicate that regardless of land use, the C balance of tropical peatlands can be sensitive to climate shifts and patterns of dry season precipitation. Although there is no consensus on how climate change will impact Southeast Asia, it is generally thought that both wet and dry seasons may intensify (Li et al. 2007). Even if total precipitation increases, HPMTrop model results presented here indicate that dry season intensification could lead to natural peat C release, though likely at much lower rates than those arising from land-use conversion.

### Management impacts on peat C dynamics

Simulated conversion of peatlands to oil palm, with an initial clearing fire burning 20 cm of surface peat, resulted in a C loss of ~300 Mg C ha^−1^ by the end of the first 25-year rotation for moderate and wet climate regimes. Carbon losses were only slightly higher under the drier GFDL climate, ranging from 320 to 330 Mg C ha^−1^. The modeled C losses, ranging from 12.4 to 13.2 Mg C ha^−1^ year^−1^ (or about 50 Mg CO_2_ ha^−1^ year^−1^; Fig. 4), are slightly larger than the IPCC default emission factor of 11 Mg C ha^−1^ year^−1^ for oil palm cultivation on drained peatland (Drösler et al. 2014).

HPMTrop simulations illustrate the potential impacts of a drier climate and increased fire frequency on peat C loss when land is cleared and abandoned (scenario 2). The tenfold difference in peat C loss between the wet MIROC climate the dry GFDL climate was due to the much higher probability of recurrent stochastic peat fire under the more intense GFDL dry season (Table 2; Page et al. 2009). Under the wet MIROC climate, simulated C losses from cleared and abandoned peatlands were about one third of those from one rotation of oil palm cultivation, whereas drier dry season conditions of the GFDL model resulted in C losses over three times greater than those of oil palm cultivation (Fig. 4). Total C loss over the 100-year simulated land degradation scenario using the GFDL model was roughly twice that lost from oil palm cultivation with fire between rotations (Table 2). Under the moderate NorESM climate, C loss from the land degradation scenario (440 Mg C ha^−1^) was similar to that of scenario 6, one 25-year oil palm rotation followed by abandonment, and less than half the loss from four oil palm rotations with burning (Table 2). For the wet MIROC climate regime, C loss for the land degradation scenario was less than any other land-use change scenario, as modeled stochastic fire probability was very low, due to low accumulated precipitation deficit and high water tables.

In 2010, the area of peatland cultivated with oil palm in Southeast Asia was estimated to be 2.1 Mha, and an additional 2.3 million ha of peatland had been cleared and left degraded (Miettinen et al. 2012; Koh et al. 2011). Combining these areas with HPMTrop simulated emissions under a moderate climate (Table 2) with one initial land-clearing burn (S6) and land degradation (S2), the combined total peat emissions from these activities is estimated to be 8.7 Gt CO_2_ over 100 years. On an annual basis, this C loss from peat alone is equivalent to about 15 % of Indonesia’s total C loss from deforestation (Harris et al. 2012). A more intense dry season under the GFDL climate model increased total emissions from oil palm cultivation and land degradation to 30.7 Gt CO_2_ over 100 years, indicating that future dry season climate and fire frequency will largely determine C emissions from converted and degraded peatlands over the twenty-first century.

### Optimizing land-use strategies for climate mitigation

Eliminating prescribed burns between oil palm rotations greatly reduced peat C losses. In total, about half the C losses could be avoided by eliminating prescribed fire between rotations (Table 2; Fig. 4), thereby reducing the C footprint of long-term oil palm cultivation. Nonetheless, simulated oil palm cultivation on peatland without burning between rotations lost 610 Mg C ha^−1^ over 100 years under the most favorable (wet) climate scenario, representing a 21 % loss of the C stock accumulated over 5000 years in just 100 years. The overall magnitude of simulated peat C loss from oil palm cultivation without fire between rotations is still several times greater than the emissions generated from the conversion of pristine tropical rainforest growing on mineral soils (IPCC 2006). Including foregone C sequestration from peat swamp forest conversion, about 2600 Mg CO_2_ ha^−1^ of emissions would be avoided over 100 years if peat swamp forest was to remain intact rather than converted to oil palm without burning, based on the conservative MIROC (wet) climate model.

The climate mitigation potential of peatland restoration following one 25-year oil palm rotation (S5) was determined by the dry season conditions of each climate model since we assumed an optimal restoration scenario, where drainage was blocked immediately to restore water tables to pristine forest levels, forest litter inputs fully recovered after 25 years (though biomass recovery would take longer), and there was no fire. Simulated restored peatlands gained 46, 110, and 140 Mg C ha^−1^ over 75 years under dry, moderate, and wet climate models, respectively (Table 2), with uptake rates highest during the middle years (Fig. 4). Therefore, emission rates of converted peatlands far exceeded sequestration rates of optimally restored peatlands for each climate regime. The highest rate of peat C accumulation, under the wet MIROC climate model, averaged 1.9 Mg C ha^−1^ year^−1^, which is well below the average 12 Mg C ha^−1^ year^−1^ of C losses from oil palm cultivation. These results indicate that the mitigation potential of peatland restoration is about 10 Mg C ha^−1^ year^−1^ less than the annual emission potential of oil palm cultivation under the most optimistic scenario. Put differently, it would take 6 years of C sequestration in restored peatlands to offset the C lost in just 1 year of oil palm cultivation, under the most optimistic climate and restoration settings. As these sequestration rates decline over time, C sequestration rates would likely decrease closer to the 0.6 Mg C ha^−1^ historical average. At historic rates of C sequestration, restored peatlands would require 20 years to offset emissions generated from one year of oil palm cultivation.

Simulated peatlands that were abandoned after one 25-year oil palm rotation (scenario 6) continued to lose C over the subsequent 75 years. Modeled C losses for abandoned peatlands were 210, 130, and 110 Mg C ha^−1^ over 75 years for dry, moderate, and wet climate models, respectively (Table 2). Carbon losses are attributed to the gradual decline of drainage ditch effectiveness rather than immediate blocking, and inclusion of a stochastic fire probability (albeit low) determined by accumulated precipitation deficit and water table depth. Interestingly, HPMTrop outputs indicate that the climate mitigation potential of peatland restoration versus abandonment and secondary succession is consistent regardless of climate, with a net balance of about 250 Mg C ha^−1^ of conserved plus gained C stock in peat over 75 years following one 25-year oil palm rotation.

Under moderate and wet dry season conditions, peatland C sequestration of intact and fully restored peatlands would likely continue close to historical averages. However, results from HPMTrop simulations must be interpreted with caution as the model does not include peat subsidence and its possible effects on drainage, water table dynamics, and in turn, forest regeneration and productivity which drive peat formation.

Results from the HPMTrop model indicate that even under the most optimistic scenario, 75 years of peatland restoration is not sufficient to offset emissions generated from one 25-year rotation of oil palm. Even after 75 years of hydrological and forest restoration, net C emissions to the atmosphere were 120 Mg C ha^−1^ under the wet climate model; this is almost the same amount of emissions generated from the conversion of lowland tropical rainforest on mineral soil. Restoring that 120 Mg C ha^−1^ in peat would take about 100 more years, if the climate stayed wet, and substantially longer if the climate dried. Nevertheless, peatland restoration following one rotation of oil palm could avoid approximately 1000 Mg C ha^−1^ of C emissions (or about 3700 Mg CO_2_ ha^−1^) under a moderate climate, compared with continuing oil palm cultivation with a prescribed burn between rotations. Therefore, the climate mitigation potential of peatland restoration is extremely high and could substantially contribute to Indonesia’s newly stated goal of reducing total greenhouse gas emissions 26 % by 2030. Avoided conversion and the removal and restoration of oil palm plantations on peatlands would be highly effective climate mitigation strategies and reduce global emissions from land use.

### Model limitations

The application of HPMTrop to simulate C dynamics of scenarios presented here is intended to illustrate the magnitude of net C gains and losses in hypothetical (yet realistic) peatlands, rather than accurately quantify emissions from actual landscapes. However, HPMTrop could be used to quantify actual C dynamics if specific site based data were available to parameterize the model. Field data and long-term measurements of peat swamp forest dynamics are lacking, therefore several assumptions were necessary to parameterize the model to the land-use scenarios considered. These include general assumptions about fire frequencies (values used represent low-medium-high frequencies), time necessary for litter inputs of restored forest to reach mature forest levels (a linear increase of 25 years), and immediate return of water table depth to pre-disturbance levels for restored peatlands. Additional field data from a range of peatland systems is needed to further improve the accuracy of HPMTrop simulations in the future.

Fire occurrence on tropical peatlands is much higher in dry years than wet years, and also much higher in drained and disturbed peatlands than undisturbed swamp forests (Miettinen et al. 2011b). However, quantitative fire probabilities for disturbed (and undisturbed) tropical peatlands are not well known. In scenarios with stochastic fire (S2 and S6) we used ad hoc high and low fire probabilities that were a function of dry season precipitation deficits to evaluate the possible importance of fire relative to the decomposition pathway. These values are highly uncertain, and the results should be interpreted in very general terms, rather than absolute predictions of future C emissions. For example, if fire occurrence rates are high, peat C loss rates will be much greater than if fire occurrence rates are low, and this will likely depend on interactions between fire management strategies and future climate patterns.

Observed C accumulations vary significantly across peatlands in insular Southeast Asia (e.g., Dommain et al. 2011). This variability is likely a consequence of a number of factors acting over the millennia of peat accumulation (climate and climate variability, topographical and hydrological setting, sea-level change, vegetation productivity and disturbance dynamics) (Dommain et al. 2016). HPMTrop peat accumulation through the Holocene varies somewhat due to a stochastic representation of climate (Fig. 3), but much less than observed in the field across the region, at least in part because it does not include site-specific setting characteristics. The degree to which this affects the simulation outcomes presented here is uncertain. The modeled peat C loss rate with drainage and oil palm cultivation is similar to the IPCC default rate (Drösler et al. 2014) but lower than those reported by Carlson et al. (2015) who calculated C emissions from drained peatlands using relationships between water table depth and peat decomposition. In all but one of the simulations, peat loss is almost exclusively from the upper 1–2 m, so the simulated results would be very similar for contemporary peatlands regardless of peat depth.

Restoration of tropical peatlands converted to agricultural production is complex, difficult, expensive, and will require long-term commitments (Dommain et al. 2016, in press). Data for secondary peat swamp forest dynamics are lacking in published literature; therefore, we used rapid (25 years) vegetation NPP and litterfall recovery in the restoration scenarios to be conservative. The optimistic recovery of litter production in S5 and, to a lesser extent, S6 probably put an upper bound on restoration C sequestration rates. Even so, C recovery for one 25-year oil palm rotation would take centuries under favorable climatic conditions, so the basic conclusion that it is a very slow process is justified.

## Conclusions

The scenarios presented here were developed as very general representations of a range of possible land-use futures for tropical peat swamp forests in Southeast Asia. Very few data are available from tropical peat swamp forests to parameterize model algorithms for fire frequencies or forest litter production recovery post-disturbance. The results of these scenarios, therefore, should be viewed as likely C balance trajectories, not accurate projections of future C balances in real systems. Nonetheless, several very general, but important conclusions can be drawn from these simulation results which inform global climate mitigation strategies for tropical peatlands.

First, large-scale emissions are avoided if peatlands remain intact, regardless of peat depth. Our results suggest that over 100 years, about 4600 Mg CO_2_ ha^−1^ of total peat emissions are avoided if peatlands are left intact rather than converted to oil palm, under a moderate climate. Second, fire management is an important consideration for climate mitigation strategies targeting peatlands. Eliminating prescribed surface fires between oil palm rotations reduced total emissions 52 %, avoiding approximately 2000 Mg CO_2_ ha^−1^ of emissions over 100 years. Fire prevention is also essential to conserve C stocks of abandoned peatlands. Simulated high fire frequency and peat decomposition under an intensified dry season resulted in total peat loss and 10,600 Mg CO_2_ ha^−1^ total emissions. Under a moderate dry season with intermediate fire frequency, total net emissions were 1600 Mg CO_2_ ha^−1^ from peat decomposition and burning for simulated peatlands that were cleared and abandoned. Simulated restored peatlands with fire prevention and rewetting maintained net C sequestration over the 75 years following oil palm cultivation. However, it is worthy to note that net C losses from 25 years of oil palm cultivation followed by 75 years of restoration were still substantial (120 Mg C ha^−1^; 440 Mg CO_2_ ha^−1^), even under optimum restoration and wet climate conditions. Third, simulated peatland restoration after 25 years of oil palm cultivation results in about 250 Mg C ha^−1^ of conserved plus gained C stock over 75 years compared with abandonment after one rotation without restoration, regardless of climate. Finally, under a moderate climate, retiring oil palm plantations after one 25-year rotation and restoring the peatland would avoid about 1700–3600 Mg CO_2_ ha^−1^ over 75 years, depending on the assumed use of fire between rotations in the “business as usual” scenario.

Rates of peat C loss due to disturbance and land-use change are much higher than rates of peat C recovery following restoration. This is particularly true if drainage and/or burning are components of the land-use management. For example, peat C losses over 100 years with four oil palm rotations with burning had taken about 3000 years to accumulate. This implies that offsetting land-use generated peat C losses will require either much larger areas of restoration or protection, or will take much longer than the duration of the land-use impact. Land-use and disturbance impacts are an immediate risk for near-surface peat, indicating that these impacts are independent of peat depth. Peat deeper than about 2 m is probably only at risk in cases of extreme burning and drainage, or persistent land-use impacts over many decades. This implies that restricting land-use activities to shallow peats will not reduce near-term impacts and C emissions, and that mapping peatland distribution and the extent of peatland disturbance are more important for accurate C accounting than mapping peat depths. Recurrent peat fire is a dominant C loss pathway, particularly when lands are left vulnerable to fire under dry conditions. Therefore, in addition to rewetting, improved fire management and prevention are essential to avoid emissions and conserve global tropical peat C stocks.
